# Erucin Exerts Anti-Inflammatory Properties in Murine Macrophages and Mouse Skin: Possible Mediation through the Inhibition of NFκB Signaling

**DOI:** 10.3390/ijms141020564

**Published:** 2013-10-15

**Authors:** Han Jin Cho, Ki Won Lee, Jung Han Yoon Park

**Affiliations:** 1 Department of Food and Nutrition, Hallym University, Chuncheon 200-702, Korea; E-Mail: hanjini@hallym.ac.kr; 2 Institute of Green Bio Science & Technology Seoul National University, Pyeongchang 232-916, Korea; E-Mail: kiwon@snu.ac.kr; 3 Advanced Institutes of Convergence Technology, Seoul National University, Suwon, Gyonggi-do 443-270, Korea; 4 WCU Biomodulation Major, Department of Agricultural Biotechnology and Center for Food and Bioconvergence, Seoul National University, Seoul 151-921, Korea

**Keywords:** erucin, inflammation, mouse skin, murine macrophages

## Abstract

Erucin, an isothiocyanate, is a hydrolysis product of glucoerucin found in arugula and has recently been reported to have anti-cancer properties in various cancer cells. In this study, we assessed the anti-inflammatory effects of erucin and the underlying mechanisms, using lipopolysaccharide (LPS)-stimulated RAW 264.7 murine macrophages and 12-*O*-tetradecanoylphorbol-13-acetate-treated mouse skin. In RAW 264.7 cells, erucin (2.5, 5 μmol/L) inhibited LPS-induced production of nitric oxide and prostaglandin E_2_. Erucin inhibited LPS-induced degradation of the inhibitor of κBα and translocation of p65 to the nucleus and, subsequently, reduced LPS-induced nuclear factor κB (NFκB) DNA binding activities, as well as the transcriptional activity of NFκB, leading to the decreased expression of NFκB-target genes, including tumor necrosis factor-α, interleukin (IL)-6, IL-1β, inducible nitric oxide synthase (iNOS) and cyclooxygenase (COX)-2, as well as transcriptional activity of iNOS and COX-2. In mice, erucin (100, 300 nmoles) treatment significantly inhibited phorbol ester-induced formation of ear edema and expression of iNOS and COX-2 proteins. These results indicate that erucin exerts a potent anti-inflammatory activity by inhibiting the pro-inflammatory enzymes and cytokines, which may be mediated, at least in part, via the inhibition of NFκB signaling.

## 1. Introduction

Inflammation is the primary response of living tissues to injury and infection and plays an important role in both innate and adaptive immunity. However, aside from these positive roles of inflammation, unresolved and recurrent inflammation is associated with a variety of diseases, including cancer, type 2 diabetes, obesity, cardiovascular diseases and neurodegenerative diseases [1]. Since inflammatory cells produce inflammatory mediators (reactive oxygen/nitrogen species and cytokines) which can induce mutation in DNA [2,3], excessive inflammatory response is a cause of cancer. Therefore, prevention and/or inhibition of inflammation are important for the prevention of these chronic degenerative diseases, and it is important to identify anti-inflammatory dietary compounds and to explore their underlying mechanisms of actions.

**Figure 1:**
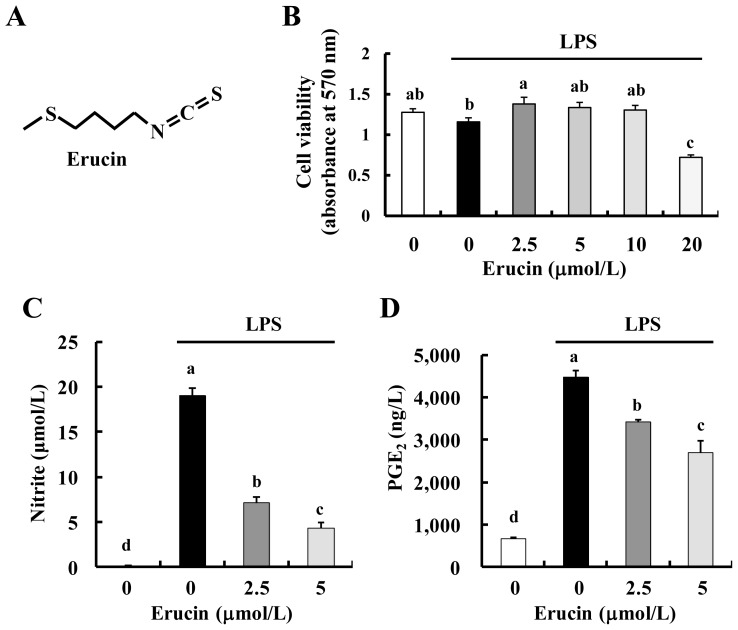
Erucin decreases lipopolysaccharide (LPS)-induced NO and prostaglandin E_2_ (PGE_2_) production in RAW 264.7 cells. (**A**) Structure of erucin; (**B**–**D**) RAW 264.7 cells were treated with various concentrations of erucin in the presence of LPS. Viable cell numbers were estimated by the MTT Assay (**B**); The 24-h-conditioned media were collected for the estimation of NO (**C**) and PGE_2_ (**D**) concentrations. Each bar represents the mean ± SEM (*n* = 4). Means without a common letter differ (*p* < 0.05).

Consumption of cruciferous vegetables is associated with several health benefits against cancer, heart health, diabetes, asthma and Alzheimer’s disease [4,5]. These benefits of cruciferous vegetables have been attributed to their containing various bioactive compounds (sulfur-containing compounds, indole derivatives and phenolic compounds). Among these compounds, isothiocyanates (sulfur-containing compounds and produced by enzymatic conversion of glucosinolates) have been shown to exert various biological effects, including anti-inflammatory properties [6]. For example, benzyl isothiocyanate (BITC), phenethyl isothiocyanate (PITC) and sulforaphane (SFN) have been found to possess anti-inflammatory activities [7–10]. Erucin, 4-(methylthio)butyl isothiocyanate (structure in Figure 1A), is a hydrolysis product of glucoerucin, which is found in arugula, and an *in vivo* reduction product of SFN. Erucin has been reported to exert chemopreventive effects by various mechanisms, including alteration of phase I, II and III enzymes, regulation of cell proliferation by induction of cell cycle arrest and apoptosis and downregulation of androgen receptor signaling [11].

The inflammatory responses are mediated by various types of cells, including lymphocytes, macrophages, dendritic cells and proliferating fibroblasts of the connective tissue [12]. During inflammation, circulating monocytes migrate into inflamed tissues and differentiate into macrophages. The activation of macrophages results in the release of pro-inflammatory cytokines, such as tumor necrosis factor-α (TNF-α), interleukin (IL)-6 and IL-1β and the production of nitric oxide (NO) by inducible nitric oxide synthase (iNOS) [13]. Prostaglandin E_2_ (PGE_2_), a key mediator of immunopathology, is also produced by cyclooxygenase-2 (COX-2) in activated macrophages [14].

Nuclear factor κB (NFκB), a transcription factor, plays a critical role in multiple biological processes, including inflammation, cell proliferation and apoptosis. It is well known that NFκB regulates the transcription of multiple genes associated with inflammatory responses, such as iNOS, COX-2, TNF-α, IL-1β and IL-6 [15]. Therefore, the inhibition of the NFκB pathway could be a good strategy to prevent and/or cure inflammation-associated diseases, and numerous inhibitors of NFκB, including natural products, have been reported through a wide variety of studies [16,17].

In this study, we explored the anti-inflammatory effects of erucin and the underlying mechanisms using lipopolysaccharide (LPS)-stimulated macrophages. Our results indicate that erucin decreases the expression of inflammatory mediators (iNOS and COX-2) and pro-inflammatory cytokines (TNF-α, IL-1β and IL-6) through the inhibition of NFκB signaling. We also demonstrate the potent *in vivo* anti-inflammatory effects of erucin in 12-*O*-tetradecanoylphorbol-13-acetate (TPA)-treated mouse skin.

## 2. Results and Discussion

### 2.1. Erucin Decreases LPS-Induced Production of NO and PGE_2_ in RAW 264.7 Cells

In order to determine non-cytotoxic concentrations of erucin for RAW 264.7 cells, we first treated the cells with various concentrations of erucin for 24 h. Cell viability was not affected by erucin up to the concentration of 10 μmol/L (Figure 1B). In order to evaluate the anti-inflammatory effects of erucin in LPS-stimulated cells, 24-h-conditioned media were collected and assayed for NO by the Griess reagent system and for PGE_2_ by ELISA. Erucin treatment decreased LPS-induced production of NO and PGE_2_ in a dose-dependent manner (Figure 1C,D).

**Figure 2:**
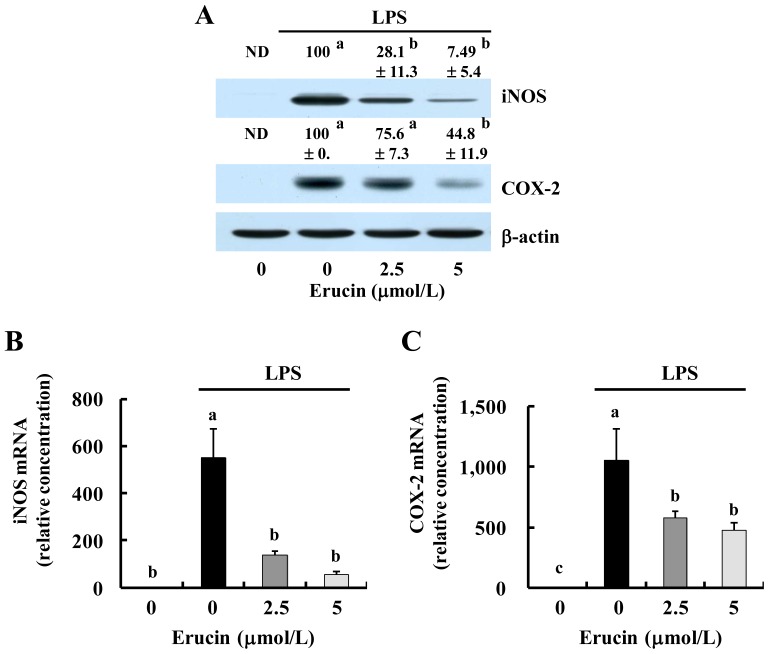

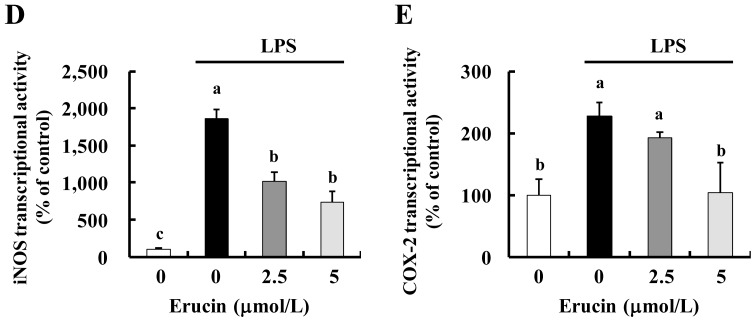
Erucin decreases LPS-induced expression of inducible nitric oxide synthase (iNOS) and cyclooxygenase (COX)-2 in RAW 264.7 cells. RAW 264.7 cells were treated with various concentrations of erucin in the presence of LPS. (**A**) Cell lysates were subjected to Western blotting with their relevant antibodies. The relative abundance of each band to their own β-actin was quantified, and the LPS control levels were set at 100%. The adjusted mean ± SEM (*n* = 3) of each band is shown above each blot; (**B**,**C**) Total RNA was isolated, and real-time RT-PCR was performed; (**D**,**E**) RAW 264.7 cells were transfected with the murine iNOS or COX-2 reporter gene construct. The transfected cells were treated with various concentrations of erucin in the presence of LPS. Cell lysates were prepared to determine luciferase activity. Each bar represents the mean ± SEM (*n* = 4). Means without a common letter differ (*p* < 0.05). ND: not detected.

We next investigated whether erucin inhibits the expression of iNOS and COX-2. The results of Western blot analysis revealed that LPS increased the protein expression of iNOS and COX-2, which was decreased by erucin treatment (Figure 2A). The levels of iNOS and COX-2 mRNAs were changed in parallel with those of their corresponding proteins (Figure 2B,C). Erucin treatment significantly inhibited LPS-induced iNOS and COX-2 transcriptional activity (Figure 2D,E). These results indicate that erucin decreases LPS-induced NO and PGE_2_ production through the downregulation of iNOS and COX-2 expression.

### 2.2. Erucin Decreases LPS-Induced Production of TNF-α, IL-6 and IL-1β in RAW 264.7 Cells

**Figure 3:**
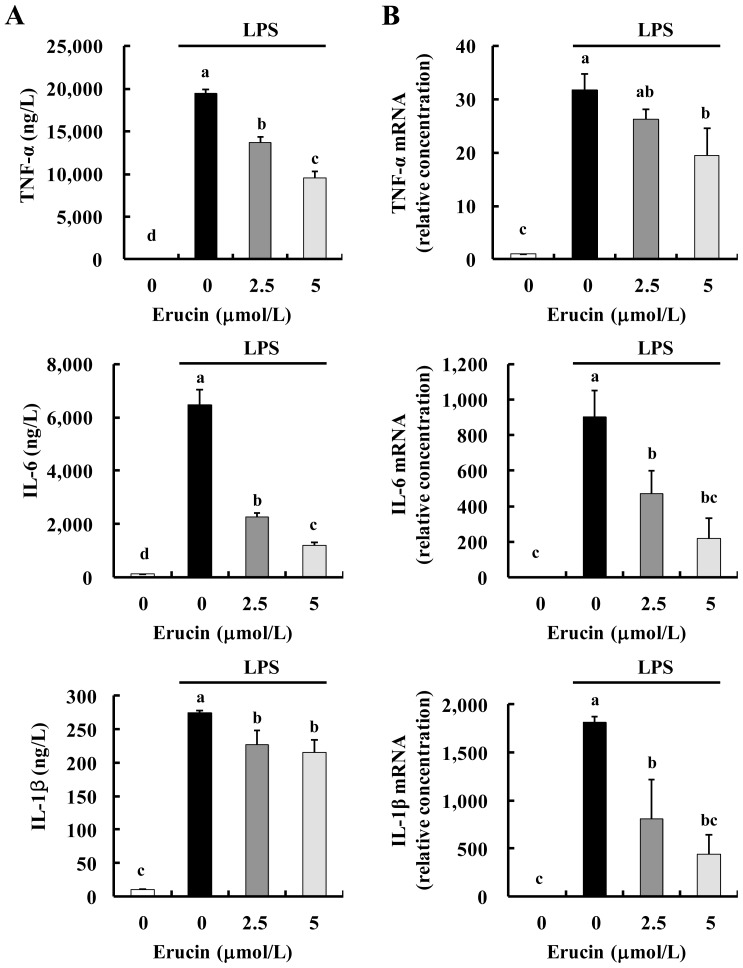
Erucin decreases LPS-induced TNF-α, IL-6 and IL-1β production in RAW 264.7 cells. RAW 264.7 cells were treated with various concentrations of erucin in the presence of LPS. (**A**) The 24-h-conditioned media were collected for the estimation of TNF-α, IL-6 and IL-1β concentrations; (**B**) Total RNA was isolated and real-time RT-PCR performed. Each bar represents the mean ± SEM (*n* = 4). Means without a common letter differ (*p* < 0.05).

We next examined whether erucin decreases the expression of pro-inflammatory cytokines. The amounts of TNF-α, IL-6 and IL-1β released into conditioned media were increased by LPS treatment, and the increases were inhibited by erucin treatment (Figure 3A). Additionally, results of real-time RT-PCR revealed that erucin inhibited LPS-induced mRNA expression of TNF-α, IL-6 and IL-1β (Figure 3B).

### 2.3. Erucin Inhibits LPS-Induced Activation of NFκB Signaling in RAW 264.7 Cells

**Figure 4:**
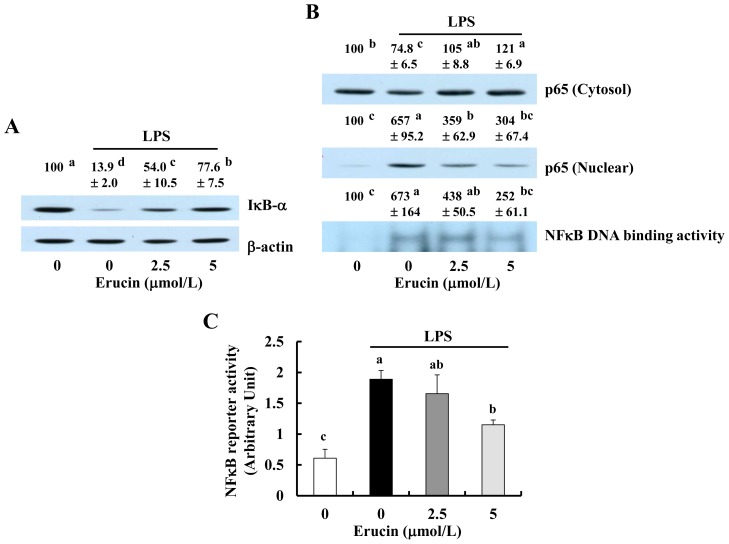
Erucin inhibits LPS-induced activation of NFκB signaling in RAW 264.7 cells. (**A**,**B**) RAW 264.7 cells were treated with various concentrations of erucin for 30 min. LPS was then added and incubated for another 20 min. (**A**) Total cell lysates were subjected to Western blotting with their relevant antibodies; (**B**) Cytosolic fractions and nuclear extracts were prepared for Western blotting with an anti-p65 antibody and Electrophoretic Mobility Shift Assay (EMSA). The relative abundance of each band was quantified, and the control levels were set at 100%. The adjusted mean ± SEM (*n* = 3) of each band is shown above each blot; (**C**) RAW 264.7 cells were transfected with NFκB-luciferase reporter plasmid. The transfected cells were treated with various concentrations of erucin in the presence of LPS. Cell lysates were prepared to determine luciferase activity. Each bar represents the mean ± SEM (*n* = 4). Means without a common letter differ (*p* < 0.05).

As NFκB regulates the expression of genes encoding iNOS, COX-2, TNF-α, IL-6 and IL-1β [18,19], we next determined whether erucin inhibits NFκB signaling. Upon stimulation, inhibitors of NFκB (IκB)-α are phosphorylated and degraded, and the NFκB proteins in the cytoplasm are liberated from IκB and translocated to the nucleus, where they bind to the promoter regions of NFκB-responsive genes [15]. LPS treatment reduced the levels of IκB-α, and erucin suppressed the LPS-induced reduction in IκB-α (Figure 4A). The levels of cytosolic p65 protein were reduced by LPS treatment, which was suppressed by erucin, whereas the levels of nuclear p65 were markedly increased by LPS, and this increase was suppressed by erucin treatment. Additionally, NFκB DNA binding was markedly increased by LPS, which was suppressed by erucin pre-treatment (Figure 4B). Furthermore, erucin inhibited the transcriptional activity of NFκB in LPS-stimulated cells (Figure 4C).

### 2.4. Erucin Decreases TPA-Induced Edema Formation in a Mouse Inflammation Model

**Figure 5:**
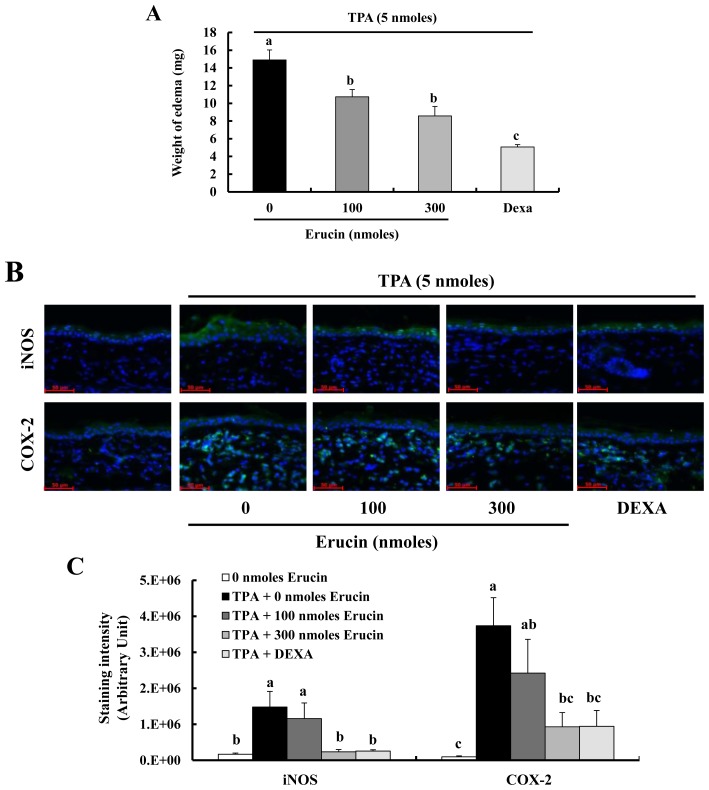
Erucin decreases 12-*O*-tetradecanoylphorbol-13-acetate (TPA)-induced edema formation in a mouse inflammation model. Erucin was topically applied to the mouse ear 30 min prior to the topical application of TPA. (**A**) The weights of 6-mm diameter ear punch samples were measured 4 h after TPA treatment. Each bar represents the mean ± SEM (*n* = 4); (**B**) Ear sections were stained with their relevant antibodies, as described in the Experimental section. Photographs of immunofluorescent staining (scale bar = 50 μm), which are representative of four different animals, are shown; (**C**) Quantification was performed by calculating the staining intensities using ImageJ. Each bar represents the mean ± SEM (*n* = 4). Means without a common letter differ (*p* < 0.05). DEXA: dexamethasone.

We next tested the *in vivo* anti-inflammatory effect of erucin using the TPA-induced mouse ear edema model. Female ICR mice were treated (in the left ear) with various doses of erucin 30 min before the application of TPA. The application of erucin to mouse ear significantly inhibited TPA-induced edema formation (Figure 5A). Immunofluorescent staining revealed that erucin pre-treatment inhibited TPA-induced iNOS expression in ear epidermis (Figure 5B,C). Using a similar skin inflammation model, we have previously reported that TPA treatment increases the infiltration of inflammatory cells in mouse skin [20]. TPA treatment induced the expression of COX-2 in infiltrating inflammatory cells, and this expression was suppressed by erucin pre-treatment (Figure 5B,C).

### 2.5. Discussion

Cruciferous vegetables, including cabbage, broccoli, mustard and arugula, are rich sources of glucosinolates, sulfur-containing compounds. Recently, glucosinolate metabolites have generated a great deal of interest, due to their biological effects, including anti-inflammatory effects [7–10], as well as anti-cancer activity [4,5,21]. For example, BITC (derived from glucotropaeolin) and PITC (derived from gluconasturtiin) have been found to possess anti-cancer [22,23] and anti-inflammatory activities [7,8]. Additionally, SFN, an isothiocyanate derived from hydrolysis of glucoraphanin, is a well-known chemopreventive compound [23] and anti-inflammatory agent [9,10]. As the inflammation mediators and cellular effectors are important constituents of the tumor microenvironments, inflammation has been incriminated in the development and progression of a variety of human cancers [24]. Thus, it can be postulated that the inhibition of inflammation is an important strategy for the inhibition of cancer development and progression by these compounds. Erucin, the sulfide analog of SFN, has been shown to exhibit anti-cancer activities [11]. However, to date, the anti-inflammatory effects of erucin have not been studied in detail, except in the recent study by Yehuda and colleagues, which reported that erucin decreased the transcription of pro-inflammatory molecules (TNF-α, IL-1β and IL-12) in THP-1 human acute monocytic leukemia cells, which were treated with LPS [25]. In the present study, we demonstrate that erucin exerts potent anti-inflammatory properties in LPS-stimulated macrophages, which is mediated through the inhibition of NFκB signaling. We also show that erucin at very low doses (100 and 300 nmoles) effectively inhibits inflammatory responses in mouse skin, suggesting that this compound has the potential to be used as an anti-inflammatory agent.

In the present study, we have demonstrated that 2.5–5 μmol/L and 100–300 nmol of erucin exert anti-inflammatory effects in macrophages and in mice, respectively. In animal studies, Abbaoui and colleagues recently showed that oral administration of erucin (7.375 μmol/25 g mice) effectively decreased tumor growth in a xenograft model of bladder cancer [26]. Erucin is a hydrolysis product of glucoerucin found in arugula and broccoli sprouts. It has been reported that when human subjects consumed 40 g of broccoli sprouts (containing 71 μmol glucoerucin), plasma erucin peaks were reached 3 h after the consumption, and the plasma concentrations of erucin were approximately 1 μmol/L [27], indicating that glucoerucin in food is rapidly metabolized and erucin is quickly absorbed into the blood. Together, these results indicate that the bioavailability of erucin is high and erucin is effective at a low dose in humans and mice. Additionally, erucin may be generated in the body from the consumption of SFN precursors, because there is evidence for interconversion between SFN and erucin in humans [27,28]. For example, erucin metabolites were found in urine samples of subjects consuming broccoli and red and white cabbage (not contain glucoerucin), and the ratio of glucoerucin/glucoraphanin in the broccoli sprouts and that of erucin/SFN metabolites in the plasma of subjects were different. However, future studies are needed to determine the efficiency of interconversion between SFN and erucin.

NFκB, an inducible transcription factor, regulates the expression of a large number of genes that are involved in the regulation of inflammation [12]. For example, inducible enzymes (iNOS and COX-2) and pro-inflammatory cytokines (including TNF-α, IL-6 and IL-1β) were regulated by NFκB activation [16,18,19]. In the present study, we observed that erucin: (1) inhibits the degradation of IκB-α and the transcriptional activity of NFκB; (2) decreases NO and PGE_2_ production through inhibition of iNOS and COX-2 expression; and (3) decreases the secretion and mRNA expression of TNF-α, IL-1β and IL-6 in LPS-stimulated macrophages. Furthermore, treatment with very low doses (100 and 300 nmoles) of erucin decreases the formation of edemas and the expression of iNOS and COX-2 proteins in TPA-treated mouse skin (Figure 5). These results indicate that erucin inhibits the expression of pro-inflammatory enzymes and the secretion of cytokines, which is mediated via the inhibition of NFκB signaling.

The NFκB family consists of five members, p65 (RelA), p50, RelB, p52 and c-Rel. NFκB is expressed in all cell types, including macrophages, dendritic cells and fibroblasts, as the main sites of inflammation. The different NFκB pathways (canonical and non-canonical) are distinguished by how they are stimulated and regulated by mediators and have different functions [12]. In the canonical NFκB pathway, the NFκB proteins (p65 and p50) are localized in the cytoplasm by IκB. Upon activation by stimuli, such as LPS, IκB kinase phosphorylates IκB protein, thereby leading to the degradation of IκB by the 26S proteasome. The liberated NFκB proteins are then translocated to the nucleus, where they bind to the promoter regions of NFκB-responsive genes, resulting in increased gene expression involved in the pro-inflammatory response [12,15]. In the present study, using murine macrophages, we clearly demonstrate that erucin inhibits LPS-induced degradation of IκB-α, translocation of p65 from the cytosol to nucleus and NFκB DNA biding activity and NFκB reporter activity (Figure 4). Results from our laboratory, as well as those from other laboratories indicate that the inhibition of NFκB signaling is one of the mechanisms by which other isothiocyanates (BITC, PITC and SFN) exert anti-inflammatory properties [7–10]. Together, these results indicate that erucin exerts anti-inflammatory properties by mechanisms similar to those of BITC, PITC and SFN. Future study is needed to explore whether mechanisms other than the inhibition of NFκB signaling are involved in the anti-inflammatory effect of erucin.

## 3. Experimental Section

### 3.1. Materials

The reagents were acquired from the following suppliers: erucin, LKT Laboratories (Saint Paul, MN, USA); 3-[4,5-dimethylthiazol-2-yl]-2,5-diphenyltetrazolium bromide (MTT), LPS, TPA and anti-β-actin antibody, Sigma (St Louis, MO, USA); antibodies against iNOS and COX-2 for Western blot analysis, BD Transduction Laboratories (Palo Alto, CA, USA); anti-NFκB p65 antibody, Santa Cruz Biotechnology (Santa Cruz, CA, USA); and anti-IκB-α antibody, Cell Signaling Technology (Beverly, MA, USA).

### 3.2. Cell Culture and MTT Assay

The RAW 264.7 cell line was purchased from the American Type Culture Collection (Manassas, VA, USA) and was maintained in Dulbecco’s Modified Eagle Medium (DMEM) containing 10% fetal bovine serum (FBS), 100 kU/L penicillin and 100 mg/L streptomycin. To examine the effects of erucin on cell viability, the cells were plated in 24-well plates at a density of 50,000 cells/well with DMEM containing 10% FBS. Cells were serum-deprived for 24 h in DMEM containing 1% FBS and were then treated with various concentrations of erucin in the presence of LPS (1 mg/L) for 24 h. Viable cell numbers were then estimated via MTT Assay.

### 3.3. NO, PGE_2_ and Cytokine Assays

The RAW 264.7 cells were treated with erucin and LPS as described above, and the 24-h-conditioned media were collected. The concentrations of NO and PGE_2_ were measured using the Griess reagent system (Promega, Madison, WI, USA) and PGE_2_ assay kit (R & D Systems, Minneapolis, MN, USA), respectively. The concentrations of TNF-α, IL-6 and IL-1β were measured using ELISA kits (eBioscience, San Diego, CA, USA).

### 3.4. Western Blot Analysis and Electrophoretic Mobility Shift Assay (EMSA)

Total cell lysates, cytosolic fractions and nuclear extracts were prepared, and Western blot analyses were performed as described previously [29]. For the Electrophoretic Mobility Shift Assay (EMSA), nuclear extracts were incubated with [^32^P]-labeled NFκB oligonucleotide probes (Promega) for 30 min [29]. Protein-DNA complexes were resolved by 5% non-denaturing gel, and the gels were then visualized by autoradiography. The intensity of the bands obtained from Western blot analysis and EMSA results was quantified by using ImageJ software (NIH, Bethesda, MD, USA). The control levels were set at 100%, and the adjusted mean ± SEM (*n* = 3) of each band is shown above each blot.

### 3.5. Real-Time RT-PCR

Total RNA was isolated using an RNeasy Plus Mini Kit (Qiagen, Valencia, CA, USA), and cDNA was synthesized using a Maxime™ RT PreMix (iNtRON Biotechnology, Seongnam, Korea). Real-time RT-PCR was conducted as previously described [29].

### 3.6. Luciferase Reporter Gene Assay

For reporter assays, RAW 264.7 cells were cotransfected with pGL-miNOS-1588 [30], pGL-mCOX-2-727 [31] or NFκB-luciferase reporter plasmid (Takara Bio, Otsu, Shiga, Japan) together with control reporter plasmid pRL-TK (Promega) using Nucleofector-II (Amaxa, Gaithersburg, MD, USA). The transfected cells were plated and treated with various concentration of erucin in the presence of LPS for 6 h. Luciferase assays were performed using the Dual-Luciferase Reporter Assay, system according the manufacturer’s instructions (Promega). The firefly luciferase activity was normalized to the Renilla luciferase activity.

### 3.7. Mouse Ear Edema

Female ICR mice (4 weeks of age) were purchased from Orient Bio Inc. (Gapyung, Korea) and were acclimatized to laboratory conditions at the animal research facility of Hallym University (Chuncheon, Korea). All animal experimental protocols were approved by the Animal Care and Use Committee of Hallym University (Hallym2010-07-1).

To evaluate the anti-inflammatory effect of erucin on ear edema formation, the mice were treated on the left ear with various doses (0, 100 and 300 nmoles) of erucin for 30 min. Edema was induced in the left ear via the topical application of 5 nmoles of TPA. Erucin and TPA were dissolved in 20 μL of dimethyl sulfoxide/acetone (15:85, *v*/*v*). Four hours after TPA treatment, biopsies of the left and right ears were performed using a 6-mm punch, and the individual biopsy was weighed. The amount of edema formation was calculated by subtracting the weight of the right ear (vehicle treated) from that of the left ear (treatment). Dexamethasone (50 μg) was used as a positive control.

For immunofluorescent staining, ear biopsies were fixed in 4% paraformaldehyde and were embedded in paraffin wax. Paraffin-embedded sections (5 μm) were hydrated through xylene and graded alcohol. Sections were then permeabilized with ice-cold methanol and incubated with anti-iNOS antibody or anti-COX-2 antibody (Cayman Chemicals, Ann Arbor, MI, USA). Sections were subsequently washed 4 times in TBS with 0.1% Tween 20 and were incubated with Alexa Fluor 488 goat anti-rabbit-IgG antibody (Invitrogen, Carlsbad, CA, USA). Nuclei were counterstained with 4′,6-diamidino-2-phenylindole. Fluorescent images were obtained using a Carl Zeiss AxioImager microscope (Carl Zeiss, Jena, Germany), and the fluorescence intensity was calculated using ImageJ software.

### 3.8. Statistical Analysis

Data were expressed as means ± SEM and analyzed by ANOVA. Differences between treatment groups were analyzed using Duncan’s multiple range test, utilizing SAS statistical software, version 9.2 (SAS Institute, Cary, NC, USA). Differences were considered significant at *p* < 0.05.

## 4. Conclusions

In summary, using RAW 264.7 murine macrophages, we demonstrate that erucin inhibits the LPS-induced production of NO and PGE_2_, by modulating the expression of iNOS and COX-2 proteins, as well as the secretion of pro-inflammatory cytokines (TNF-α, IL-6 and IL-1β). Additionally, we demonstrate that erucin inhibits the transcriptional activity of *iNOS* and *COX-2* and mRNA expression of iNOS, COX-2 TNF-α, IL-6 and IL-1β, as well as NFκB signaling. Furthermore, erucin suppresses inflammatory responses and inhibits the expression of iNOS and COX-2 in TPA-treated mouse skin. Recurrent and persistent inflammation is associated with a broad variety of diseases. The present results suggest that erucin can be used as a natural anti-inflammatory agent, which can help to prevent or relieve chronic inflammation-related diseases.
